# Dentoalveolar changes following extraction of mandibular primary second molars in patients with congenitally missing second premolars—a longitudinal randomized controlled trial

**DOI:** 10.1093/ejo/cjaf095

**Published:** 2025-11-19

**Authors:** Shaker Nawaia, Nameer Al-Taai, Sarah Abdul Jabbar, Ken Hansen, Julia Naoumova

**Affiliations:** Clinic of Orthodontics, Public Dental Service, Region Västra Götalandsregionen, Skövde, Sweden; Department of Odontology, Umeå University, Umeå, Sweden; Hamdan Bin Mohammed College of Dental Medicine, MBRU University, Dubai, UAE; Clinic of Orthodontics, University Dental Care, Public Dental Service, Region Västra Götaland, Gothenburg, Sweden; Clinic of Orthodontics, University Dental Care, Public Dental Service, Region Västra Götaland, Gothenburg, Sweden; Clinic of Orthodontics, University Dental Care, Public Dental Service, Region Västra Götaland, Gothenburg, Sweden; Department of Orthodontics, Institute of Odontology, Sahlgrenska Academy, University of Gothenburg, Sweden

**Keywords:** Hypodontia, Long-term outcome, interceptive extraction, Tooth movement

## Abstract

**Background:**

Early management of congenitally missing mandibular second premolars may influence craniofacial growth, yet the long-term effects of extracting the primary second molars remain unclear.

**Objectives:**

To evaluate the skeletal and dental effects of extracting mandibular primary second molars in patients with congenitally missing mandibular second premolars and to compare with historical control data.

**Trial design:**

Prospective, randomized longitudinal split-mouth trial.

**Materials and Methods:**

This longitudinal study is based on 40 patients (aged 9–12 years) with bilateral agenesis of mandibular second premolars, who were randomly assigned to either extraction or hemisection of the primary second molar. Randomization to determine the side and order of extraction or hemisection was performed using an online tool, with allocation concealment ensured by sequentially numbered, opaque, sealed envelopes prepared in advance by the author responsible for treatment assignment, who was not involved in patient care. The present study examines the overall treatment effect, regardless of the method applied in the split-mouth design. Cephalometric analyses were performed at baseline (T1) and after 4 years (T2). Dental casts were evaluated at T2 to measure residual space. A control group of 29 untreated individuals with normal occlusion was included for comparison. All measurements were conducted by an independent examiner blinded to the treatment assignment.

**Results:**

In the extraction group both the maxilla and the mandible exhibited forward growth (*P* < .05), while only minor changes were detected in the control group. Extraction caused significant retroclination of the lower incisors by 4.5° (*P* < .01) and retrusion by 1.8 mm (*P* < .01), leading to an increased overjet of 1.2 mm (*P* < .05) and overbite of 1.5 mm (*P* < .05). Compared with controls, dental changes were significantly greater in the extraction group (*P* < .05). Residual space after extraction showed no significant correlation with cephalometric changes.

**Conclusion:**

Extraction of mandibular primary second molars in cases of agenesis induces favourable dental changes without detrimental skeletal effects. This approach may be a viable option in selected patients, although caution is recommended for those with deep bite tendencies.

**Trial registration:**

The trial was registered with https://www.researchweb.org/, registration number: 967125.

## Introduction

Tooth agenesis, or the congenital absence of teeth, is one of the most common dental anomalies in children. It results from disturbances during the initiation and proliferation stages of tooth development, which can prevent the formation of dental buds [1]. The prevalence of agenesis varies depending on ethnicity, gender, and population, and when third molars are excluded, the condition affects ∼6.1%–8.4% of the global population [1–4].

In Scandinavian populations, the most frequently missing tooth is the mandibular second premolar, with a reported prevalence of (∼3%), and nearly half of these cases are bilateral [4–7]. Agenesis of the mandibular second premolars is typically diagnosed around 8–9 years of age, as these teeth may not mineralize until this developmental stage [7]. The management of second premolar agenesis involves a range of treatment strategies, often based on factors such as the patient’s age, skeletal development, occlusion, space conditions, and long-term restorative or orthodontic goals. One conservative option is to retain the primary second molars when they are healthy, functional and show no signs of root resorption or ankylosis [8]. However, retention is not always a long-term solution, as primary molars may become infraoccluded, resorbed, or ankylosed over time, potentially complicating future treatment [8].

An alternative approach is the extraction of the primary second molars [9–12]. This can be performed by conventional extraction or through hemisection, where the tooth is extracted in two stages, typically starting with the distal root, followed by the mesial root several months later [13, 14]. The goal of early extraction, particularly before the eruption of the permanent second molars, is to facilitate spontaneous mesial migration of the first molars and enable space closure with minimal orthodontic intervention [11, 12]. This can result in a space reduction of <2 mm, often achieved through both bodily tooth movement and mesial tipping of the adjacent teeth [12, 14].

The amount of mesial tipping and space closure following extraction depends significantly on the timing of the intervention, the patient’s individual growth pattern, and the degree of vertical facial development [13]. Hemisection is thought to preserve the buccolingual integrity of the alveolar ridge, reduce mesial tipping, and promote more parallel movement of the adjacent teeth by maintaining bone support during the early stages of movement [13]. However, recent evidence indicates that hemisection offers no significant advantage over conventional extraction in terms of space closure or tooth inclination, and both methods yield similar outcomes [14].

When spontaneous space closure is insufficient or not feasible, active orthodontic treatment may be necessary. This often involves the use of fixed appliances in combination with Class II elastics or temporary anchorage devices to close spaces effectively. In select cases, particularly when space closure is not desired or possible, restorative options such as autotransplantation of third molars, dental implants, or fixed prosthetic replacements may be considered, especially after skeletal growth is completed [9, 10].

The impact of extractions on the soft tissue profile is complex and influenced by multiple factors [15]. Recent studies have shown that premolar extraction in adolescents primarily affects the dental and soft tissue profile, leading to retraction of the anterior teeth and lips, along with an increase in the nasolabial angle, while skeletal changes remain minimal [16, 17]. Although premolar extractions have been extensively studied, the skeletal and dental effects of extracting mandibular primary second molars in patients with congenitally missing second premolars remain unexplored. As the first paper from this trial found no difference in residual space between conventional extraction and hemisection [14] in patients with congenitally missing mandibular second premolars, the present study examines the overall impact of extracting two deciduous molars—regardless of method—on dentoalveolar and skeletal changes, with a secondary aim of comparing the outcomes with historical control data.

### Subjects and methods

The study was conducted in accordance with the Declaration of Helsinki, and ethical approval was granted by the Regional Ethical Review Board at the University of Gothenburg (reg. no. 558-17). The first part of the study assessing space closure was published in 2025 [14].

### Trial design

The study was based on a single-centre, prospective, split-mouth, randomized controlled clinical trial. The present longitudinal study examines the overall treatment effect, regardless of the method applied, in the split-mouth design.

### Participants, setting, and eligibility criteria

Participants were recruited from nine public dental clinics in the Skaraborg region between 2017 and 2020. Written and oral information was given and consent was obtained before randomization. The inclusion criteria were:

- Age 9–12 years
- Bilateral agenesis of the mandibular second premolars
- Bilateral persisting deciduous second mandibular molars
- Bilateral presence and unerupted mandibular second molars

The exclusion criteria were:

- Angle Class II:2 (as mandibular extractions could potentially deepen the bite)
- Generalized spacing in the mandible (defined as ≥8 mm of spacing measured from the mesial right primary second molar to the mesial left primary second molar)
- Cleft lip and palate or other craniofacial deformities

### Sample size

The sample size was calculated for the first part of the trial aiming to detect a clinically meaningful difference of 2 mm (±1 mm) in space closure between the two extraction methods [14]. With a Type I risk of 5.0% and a Type II risk of 10%, we estimated that 30 patients would be necessary. To account for a potential 30% dropout rate, the sample size was adjusted to 40 patients.

### Randomization

For each patient, the extraction method, including the tooth selection and procedure order, was randomized using an online platform (https://www.graphpad.com/quickcalcs/randomize1.cfm) by one author (J.N.) who was not involved in patient enrolment or treatment. Allocation concealment was ensured with sequentially numbered, opaque, sealed envelopes prepared in advance by the same author.

### Intervention

**Figure 1.**
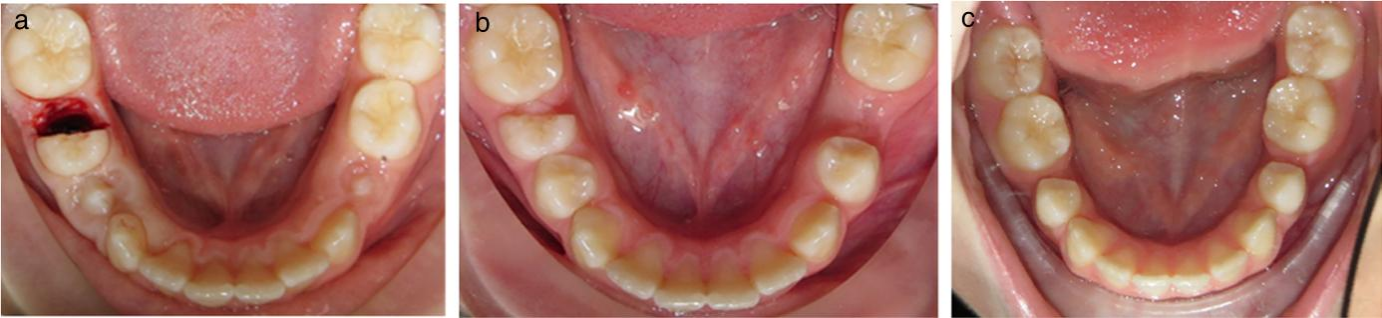
(a) Hemisection of the distal root and crown of the right primary second molar. (b) When the residual space was ≤2 mm, the mesial part of the tooth was extracted. (c) Space closure at the follow-up (T2).

Patients were randomized for either extraction or hemisection on the left or right side of the mandible (Fig. 1). *Hemisection:* The primary second mandibular molar was sectioned at the furcation using a Zekrya surgical bur. The distal root and crown were removed with extraction forceps, leaving the mesial portion exposed without surgical dressing or endodontic intervention (Fig. 1a). The mesial segment was extracted when the residual space was ≤2 mm (Fig. 1b). *Conventional extraction:* the primary tooth was removed using extraction forceps (1B).

None of the participants underwent orthodontic treatment between baseline (T1) and the follow-up assessment (T2).

### Control group

For the control group, historical data from Sweden were used, consisting of cephalometric radiographs of untreated subjects taken at 12 and 15 years of age [18]. The inclusion criteria were Angle Class I normal occlusion, normal overjet and overbite, normal transversal relation, up to 1 mm space deficiency in each jaw, and a harmonious soft tissue profile.

### Measurements

#### Residual space

Residual space was evaluated on cast models using three outcome measures: molar–premolar distance, premolar–canine distance, and canine–incisor distance. Measurements were obtained with a digital calliper accurate to 0.1 mm (Karl Hammacher GmbH, REF HSL 246-15). For spaces smaller than 1.00 mm, a manual interproximal measuring instrument was employed, featuring calibrated thicknesses of 0.10, 0.15, 0.20, 0.25, 0.30, 0.40, 0.50, and 1.00 mm (Intensiv IPR Distance Control, REF IPR-DC set).

#### Cephalometric measurements

**Figure 2.**
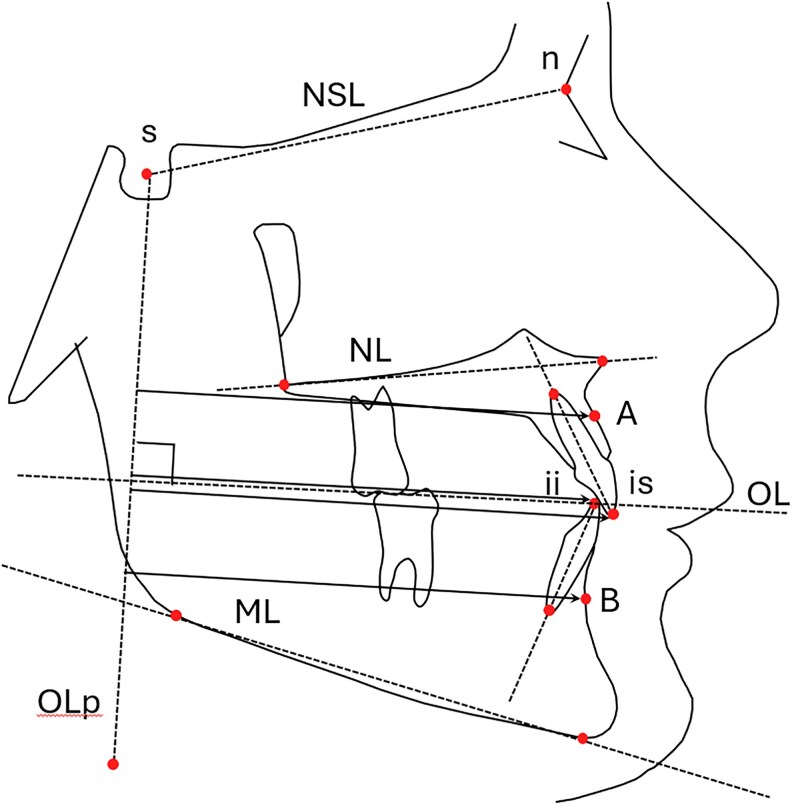
The cephalometric landmarks and lines used in the study. Cephalometric measurements: NSL: nasio-sella line, NL: maxillary plane, ML: mandibular plane, OL (occlusal line), OLp (occlusal line perpendicular), A-Olp: distance from A, A-point to OLP, is-Olp: distance from is: upper incisors to OLP, B-Olp: distance from B, B-point to OLP, ii-Olp: distance from ii, lower incisors to OLP [19]. OJ, overjet: horizontal distance between the buccal surface of the lower incisors and the palatal surface of the upper incisors. OB, overbite: vertical overlap of the upper front teeth over the lower front teeth.

Cephalometric radiographs in centric occlusion were taken at baseline (T1) and follow-up (T2). The cephalometric radiographs were taken using two different X-ray machines, Planmeca ProMax with the exposure settings of 66 kV and 10.0 mA, and Instrumentarium OC200 with the exposure settings of 66 kV and 10.0 mA. Cephalometric analysis was performed using FACAD® software (version 3.9.2.1133; Ilexis AB, Linköping, Sweden). The radiographs were calibrated using a midline magnification factor of 1.1. The cephalometric landmarks and reference lines are presented in Fig. 2. All tracings were conducted by a single examiner (S.N.).

The cephalometric variables used in the study, apart from standardized cephalometric values, are shown in Fig. 2. The occlusal line perpendicular (OLp), passing through Sella (S), was used as a reference grid (Fig. 2). Following superimposition of the Nasion-Sella line (NSL) line with Sella as the registration point, the reference grid from the initial radiograph was transferred to the T2 radiographs. Sagittal skeletal and dental changes were assessed according to the method suggested by Pancherz [19]. To evaluate quantitatively the anteroposterior position of the incisors relative to their respective skeletal bases, the following distances were calculated: (i) is-A, position of the central maxillary incisor in relation to the maxillary base (is-OLp minus A-OLp), and (ii) ii-B, position of the central mandibular incisor in relation to the mandibular base (ii-OLp minus B-OLp).

### Blinding

This was a single-blind study, with all measurements conducted by an independent examiner who was unaware of each patient’s treatment allocation.

### Error of method

Intra-observer reliability was determined using intraclass correlation coefficients (ICC) by repeating measurements on 25 randomly selected patients by the same operator (S.N.) at two separate time points, three weeks apart. Inter-observer reliability was assessed using ICC through measurements performed by a second operator (S.A.J.) on the same set of 25 radiographs.

The intra-observer ICC values for both linear and angular measurements were all above 0.859, with the lowest confidence interval (CI) at 0.868. Measurement error percentages were below 10% across all variables. Similarly, the inter-observer ICC values for linear and angular measurements exceeded 0.878, with the lowest CI being 0.709. The measurement error percentages remained below 11.2% for all variables.

### Statistics

Descriptive statistics, including mean values, standard deviations (SDs), and 95% CIs, were calculated. Paired *t*-tests were used to assess within-group differences for both the extraction and control groups, while unpaired *t*-tests were applied to compare measurements between the two groups. Pearson correlation coefficients were calculated to examine the relationship between cephalometric changes and residual extraction gaps. All statistical analyses were performed using SAS software, version 9.3 (SAS Inc., Cary, NC, USA), with a significance level set at *P* < .05.

## Results

**Figure 3.**
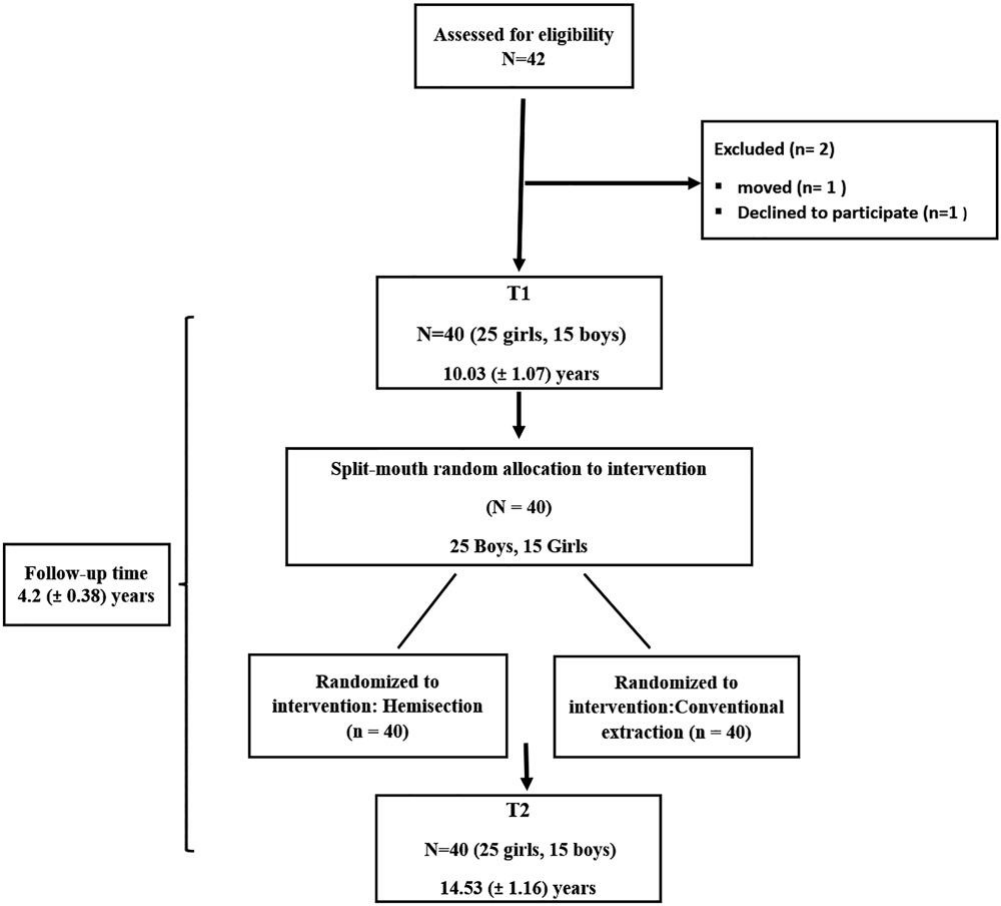
Flowchart illustrating the extraction group.

The extraction group consisted of 40 patients: 25 boys and 15 girls (Fig. 3). The mean age at T1 was 10.03 ± 1.07 years (boys: 10.5 ± 1.17, girls: 10.02 ± 0.83), and at T2, 14.53 ± 1.16 years (boys: 14.72 ± 1.25, girls: 14.2 ± 0.96). The follow-up time was 4.2 ± 0.38 years, and no patients were lost to follow-up (Fig. 3). The control group consisted of 29 untreated subjects, comprising ten boys and 19 girls, with a mean age at T1 of 12.8 ± 0.3 years and at T2 of 15.7 ± 0.4 years.

### Extraction group

**Figure 4.**
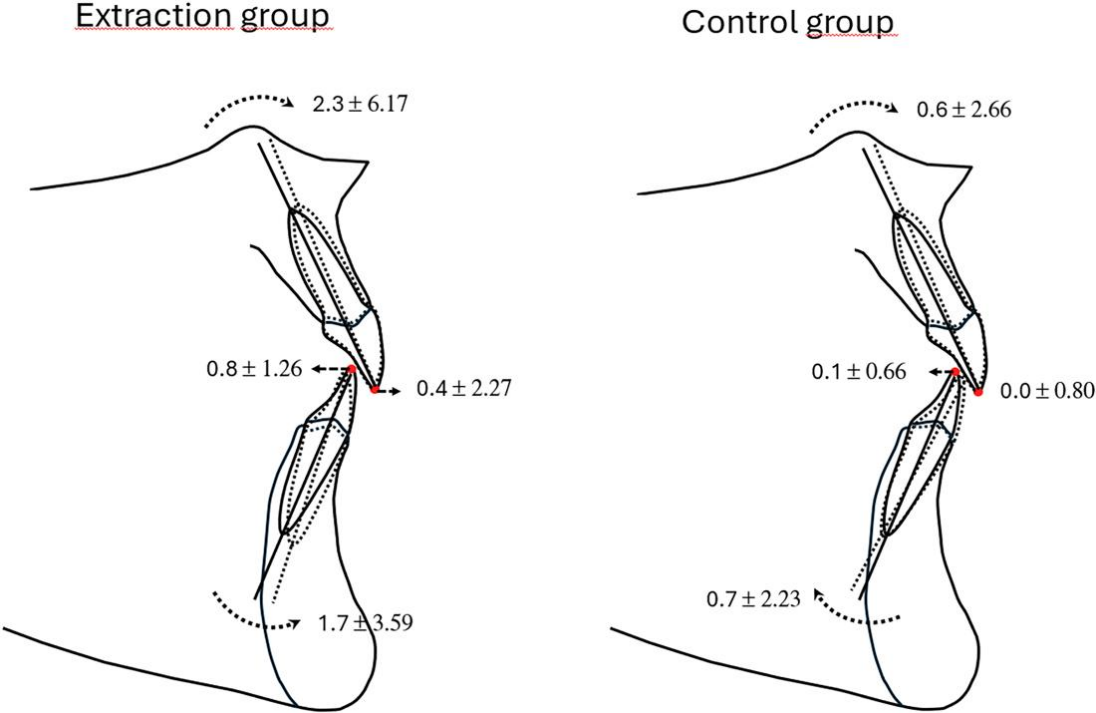
Mean changes and SD from baseline to follow-up in for incisor movements in degrees and millimetres in the extraction and control group.

**Figure 5.**
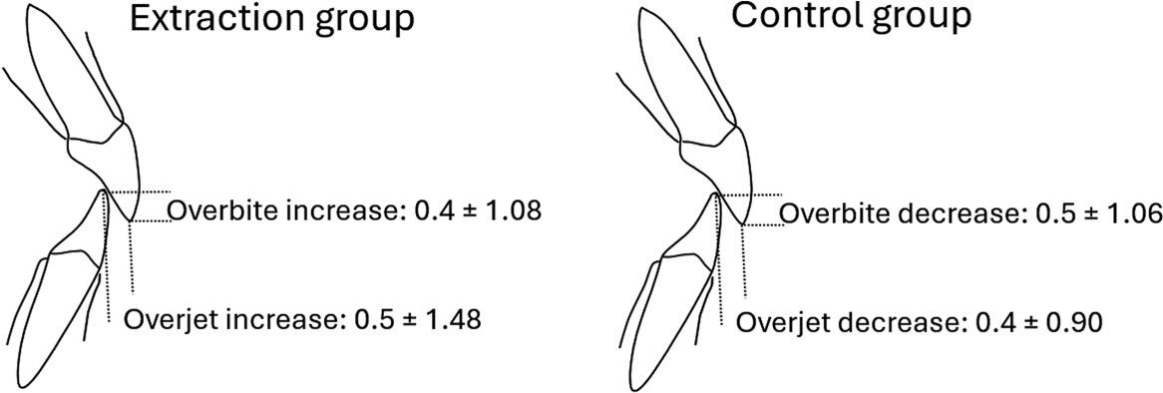
Mean changes in overbite and overjet from baseline to follow-up in the extraction and control group.

Significant changes were observed between T1 and T2 following bilateral extraction of the primary second molars. Forward growth of the maxilla and mandible was indicated by an increase in sagittal position of the maxilla in relation to the cranial base (SNA) by 1.76° ± 1.93 (*P* < .0001) and in sagittal position of the mandibel in relation to the cranial base (SNB) by 1.63° ± 2.24 (*P* < .0001), while decreased NSL/ML (−2.19° ± 2.95, *P* < .0001) and inclination of the mandibular plane to the palatal plane (NL/ML) (−1.80° ± 2.78, *P* = <.001) values suggested anterior mandibular growth. Dentally, the interincisal angle increased significantly by 5.79° ± 7.34 (*P* < .0001), accompanied by retroclination of both the upper (ILs/NL: −2.26° ± 6.17, *P* = .025) and lower incisors (ILi/ML: −1.71° ± 3.59, *P* = .004), resulting in increasing overjet by 0.55 mm ± 1.48 (*P* = .024) and overbite by 0.43 mm ± 1.02 (*P* = .01) (Table 1). Retroclination of the lower incisors was observed irrespective of growth effects, resulting in an increase in overjet and overbite (Table 1, Figs 4 and 5). No gender differences were observed between T1 and T2.

**Table 1.** Mean changes and SD of cephalometric variables at baseline (T1) and follow-up (T2) for the extraction group.

Variable	T1		T2		T2-T1		*P*-value
	Mean	SD	Mean	SD	Mean	SD	
Skeletal							
SNA (°)	81.89	3.33	83.66	4.01	1.76	1.93	<.0001***
SNB (°)	79.13	3.45	80.76	4.34	1.63	2.24	<.0001***
ANB (°)	2.89	1.53	3.12	1.66	0.22	1.19	.23
Wits (mm)	1.57	1.48	2.32	2.05	0.75	2.23	.039*
NSL/NL (°)	6.27	2.90	5.85	3.47	−0.41	2.01	.19
NSL/ML (°)	31.93	5.71	29.74	7.10	−2.19	2.95	<.0001***
NL/ML (°)	25.79	5.54	23.98	23.98	−1.80	2.78	.0002***
Dental							
ILs/NL (°)	111.40	6.91	109.13	8.19	−2.26	6.17	.025*
Ili/ML (°)	92.70	6.94	90.99	6.79	−1.71	3.59	.004**
Interincisive (°)	130.09	10.37	135.88	12.03	5.79	7.34	<.0001***
Ils/NA (°)	23.36	6.50	19.72	8.22	−3.63	6.63	.001***
ILi/NB (°)	23.77	6.75	21.50	6.50	−2.27	3.40	.0001***
is-A (mm)	6.03	2.17	6.44	3.26	0.41	2.27	.259
ii-B (mm)	2.62	1.59	1.76	1.46	−0.85	1.26	.0001***
OJ (mm)	2.63	1.06	3.18	1.64	0.55	1.48	.024*
OB (mm)	3.13	1.46	3.57	1.46	0.43	1.02	.01*

^*^ *P*\.05. **P\.01. ***P\.001. Negative values represent decreases during treatment; positive values represent increases during treatment.

At T2, the average residual mandibular space, measured bilaterally as the molar–premolar, premolar–canine, and canine–incisor distances was 9.56 ± 4.65 mm. No significant correlations were found between residual space and cephalometric variables during the observation period, except for SNA and SNB, which showed low correlations (0.22 and 0.115, respectively).

### Control group

In the control group, significant changes between T1 and T2 were limited to a reduction in vertical skeletal measurements (NSL/ML, NL/ML, relationship jaw angle between the ramus and the corpus (ML/RL), NSL/OL), indicating anterior mandibular growth. Dentally, only overjet and overbite decreased significantly by −0.38 mm ± 0.90 (*P* = .029) and −0.47 mm ± 1.06 (*P* = .022), respectively (Table 2).

**Table 2.** Mean changes and SD of cephalometric variables at baseline (T1) and follow-up (T2) for the control group.

Variable	T1		T2		T2-T1		*P*-value
	Mean	SD	Mean	SD	Mean	SD	
Skeletal							
SNA (°)	82.46	3.13	82.47	2.73	0.003	1.06	.986
SNB (°)	80.78	2.49	80.85	2.54	0.07	0.75	.590
ANB (°)	1.7	1.47	1.62	1.67	−0.07	0.98	.669
Wits (mm)	−0.94	1.89	−0.3	2.31	0.63	1.76	.062
NSL/NL (°)	6.03	2.71	6.41	2.56	0.37	1.27	.119
NSL/ML (°)	31.59	4.19	30.58	4.47	−1.006	1.16	<.0001***
NL/ML (°)	25.53	4.24	24.16	4.21	−1.36	1.3	<.0001***
Dental							
ILs/NL (°)	111.97	4.39	111.33	4.79	−0.64	2.66	.203
Ili/ML (°)	88.42	4.65	89.17	4.73	0.75	2.23	.08
Interincisive (°)	134.04	7.32	135.3	7.32	1.25	3.27	.048
Ils/NA (°)	23.46	3.93	22.45	4.66	−1.006	3.01	.082
ILi/NB (°)	20.78	5.02	20.63	5.46	−0.15	1.8	.653
is-A (mm)	5.98	1.31	5.95	1.52	−0.03	0.8	.837
ii-B (mm)	1.65	1.64	1.55	1.74	−0.1	0.66	.424
OJ (mm)	2.8	0.78	2.41	0.66	−0.38	0.9	.029*
OB (mm)	3.09	0.9	2.61	0.81	−0.47	1.06	.022*

^*^ *P*\.05. ***P\.001. Negative values represent decreases during treatment; positive values represent increases during treatment.

### Extraction versus control group

Comparisons between the groups showed significant skeletal differences: SNA and SNB angles increased in the extraction group and remained unchanged in the control group (*P* < .001 and *P* < .001, respectively), while NSL/ML decreased in both groups with a more significant reduction in the extraction group (*P* = .044).

Dental changes were more pronounced in the extraction group, with a significant increase in the interincisal angle (*P* = .002) and reductions in incisor inclination (*P* = .001) and protrusion (*P* = .004). Overjet and overbite increased in the extraction group but decreased in the control group, indicating incisor retroclination and retrusion associated with extraction (*P* = .003 and *P* = .0006, respectively) (Table 3, Figs 4 and 5).

**Table 3.** Comparison of extraction and control groups regarding changes in cephalometric variables between T1 and T2.

Variable	Extraction group			Control group			Difference			*P*-value
	Mean	SD	95% Cl	Mean	SD	95% Cl	Mean	SD	95% Cl	
Skeletal										
SNA (°)	1.76	1.93	1.14, 2.38	0.00	1.06	−0.4, 0.4	−1.76	1.63	−2.55, −0.96	<.0001***
SNB (°)	1.63	2.24	0.91, 2.34	0.07	0.75	−0.21, 0.36	−1.55	1.77	−2.42, −0.69	<.0001***
ANB (°)	0.22	1.19	−0.15, 0.6	−0.07	0.98	−0.4, 0.29	−0.3	1.11	−0.84, 0.23	.263
Wits (mm)	0.75	2.23	0.036, 1.46	0.63	1.76	−0.03, 1.31	−0.11	2.05	−1.11, 0.88	.823
NSL/NL (°)	−0.41	2.01	−1.06, 0.22	0.37	1.27	−0.1, 0.86	0.79	1.74	0, 1.64	.065
NSL/ML (°)	−2.19	2.95	−3.13, −1.24	−1.01	1.16	−1.45, −0.56	1.18	2.37	0.03, 2.34	.044*
NL/ML (°)	−1.80	2.78	−2.69, −0.91	−1.36	1.3	−1.86, −0.87	0.43	2.28	−0.67, 1.54	.436
ML/RL (°)	−1.06	5.13	−2.7, 0.58	−2.23	1.67	−2.87, −1.6	−1.17	4.06	−3.15, 0.8	.239
Dental										
ILs/NL (°)	−2.26	6.17	−4.24, −0.29	−0.64	2.66	−1.66, 0.37	1.62	5.01	−0.81, 4.06	.188
Ili/ML (°)	−1.71	3.59	−2.86, −0.56	0.75	2.23	−0.09, 1.6	2.46	3.09	0.96, 3.97	.001***
Interincisive (°)	5.79	7.34	3.442, 8.14	1.25	3.27	0.007, 2.5	−4.53	5.99	−7.45, −1.61	.002**
Ils/NA (°)	−3.63	6.63	−5.75, −1.51	−1.01	3.01	−2.15, 0.13	2.62	5.42	−0.01, 5.26	.05*
ILi/NB (°)	−2.27	3.40	−3.36, −1.18	−0.15	1.8	−0.83, 0.53	2.12	2.84	0.73, 3.51	.003**
is-A (mm)	0.41	2.27	−0.31, 1.13	−0.03	0.8	−0.33, 0.27	−0.44	1.81	−1.32, 0.43	.32
ii-B (mm)	−0.85	1.26	−1.26, −0.45	−0.10	0.66	−0.35, 0.15	0.75	1.05	0.24, 1.27	.004**
OJ (mm)	0.55	1.48	0.07, 1.02	−0.38	0.9	−0.73, −0.04	−0.93	1.27	−1.56, −0.31	.003**
OB (mm)	0.43	1.02	0.1, 0.76	−0.47	1.06	−0.88, −0.07	−0.91	1.04	−1.41, −0.4	<.0001***

^*^ *P*\.05. **P\.01. ***P\.001. Negative values represent decreases during treatment; positive values represent increases during treatment.

### Harms

No harmful effects were observed during this part of the study.

## Discussion

In the first paper from this trial, the residual space following conventional extraction and hemisection was evaluated, with findings showing no difference between the two sides [14]. Consequently, this part of the study focuses not on side-to-side comparisons, but on the overall impact of extracting two deciduous molars, regardless of the method, on dentoalveolar and skeletal changes. The main findings demonstrated that both the upper and lower incisors exhibited small but statistically significant retroclination and retrusion between T1 and T2; however, these changes were of limited clinical significance.

The increases in SNA and SNB observed in the intervention group are likely attributable to normal growth, as well as differences in age and observation period between the groups. The vertical jaw relationship (NL/ML) decreased by 1.3°, with no significant differences between groups. Similarly, the mandibular plane angle showed a slight reduction—1° in the control group and 2° in the extraction group—resulting in mild anterior mandibular rotation. These changes occurred alongside overall increases in both anterior and posterior facial heights. Such skeletal modifications are more likely related to normal growth than to the extraction of the primary second mandibular molars. Dentoalveolar changes, namely protrusion and retrusion, were assessed using Pancherz’s method, which accounts for growth by evaluating changes both within and between the jaws [19].

While the present study found no correlation between residual space and skeletal cephalometric variables, Lindqvist [11] observed a tendency towards a positive correlation between space size and ANB angle 1-year post-extraction.

The significant increase in overjet and overbite following extraction in our study was primarily due to retrusion and retroclination of the mandibular incisors, as indicated by a significant decrease in (ii-B and Ili/ML), while maxillary incisors were more stable. When the extraction group was compared with a control group, a significant difference was found in retroclination and retrusion of the lower incisors, whereas no statistically significant differences were observed in the upper incisors. These results contrast with those of Lindqvist [11], who reported minimal changes and suggested that overjet and overbite remained stable following the extraction of mandibular primary second molars. However, Lindqvist’s conclusions were based solely on the interincisal angle, without presenting specific measurements for overjet or overbite. The timing of the radiographs at follow-up was also not clearly stated. Moreover, discrepancies between the studies may be attributed to differences in patient age and sample characteristics, as Lindqvist included younger patients and cases involving compensatory extractions in the maxilla [11].

The increase in overbite observed in our study supports previous findings that associate dental extractions with vertical changes. Richardson [20] reported similar outcomes in a study of 43 patients aged 8–14, where lateral cephalometric radiographs taken before and ∼12 months after the extraction of the lower first permanent molars revealed spontaneous changes in overbite, overjet, and incisor inclination. Notably, overbite deepened in more than half of the cases, while overjet remained largely unchanged [20]. In our cohort, overbite increased by 0.4 ± 1.02 mm and overjet by 0.5 ± 1.48 mm during the follow-up period, aligning with the trend described in the literature [16]. These relatively minor changes may be considered acceptable in clinical practice, suggesting that interceptive extraction—despite leaving residual spaces—could be a viable treatment option. However, when greater vertical and sagittal control is required, fixed appliances may be necessary to guide proper occlusal development. Importantly, in patients with an already pronounced deep bite, extraction of the second primary molars should be avoided as the only measure, as it may exacerbate vertical and sagittal discrepancies. In such cases, retaining the second primary molars is advisable, given their demonstrated long-term survival and stabilizing potential, until a complete treatment plan is established [8, 21].

### Strengths and limitations

The study has several strengths, including its prospective design with a long follow-up period and the use of a blinded operator to ensure unbiased measurement of comprehensive dental and skeletal cephalometric parameters. However, some limitations should be noted: the control group was historical, resulting in unequal sample sizes between groups. Additionally, differences in age and follow-up duration, with the control group being slightly older and having a shorter follow-up, may introduce maturation bias affecting cephalometric changes during puberty. Nonetheless, the historical control group was selected from a prior Swedish study to minimize ethical concerns.

### Generalizability

The results of this study are applicable to comparable cohorts of Caucasian patients with bilateral agenesis of the mandibular second premolars, as well as to control groups of the same ethnic background.

### Clinical implications

The findings suggest that interceptive extraction of mandibular primary second molars in patients with congenitally missing second premolars results in minor dental changes, such as retroclination and retrusion of the lower incisors resulting in a slight increase in overjet and overbite. These changes are generally of limited clinical concern and support the use of extraction as a viable early interceptive treatment option. However, in cases requiring greater vertical or sagittal control—especially patients with a pronounced deep bite—extraction alone may worsen occlusal discrepancies. In such situations, retention of the primary molars or the use of fixed orthodontic appliances should be considered to maintain stability and guide proper occlusal development.

## Conclusion

Extraction of primary second molars in patients with congenitally missing mandibular second premolars resulted in lower incisor retroclination and a minor, clinically insignificant increase in overjet and overbite. No association was found between residual space and cephalometric changes. Compared with the control group, the extraction group showed somewhat more pronounced dental changes than skeletal alterations.

## Data Availability

All data generated or analysed during this study are included in this article. Further enquiries can be directed to the corresponding author.
